# Effectiveness of Homoeopathic Treatments for Sleep Disorders in Children and Adolescents: A Systematic Review According to the Principles of Evidence-Based Medicine

**DOI:** 10.3390/children13010045

**Published:** 2025-12-29

**Authors:** Kanchan Upreti, Michael Frass

**Affiliations:** 1 Dr. Kanchan Cures, New Delhi 110074, India; 2 Center for Applied Prevention, Blasewitzer Str. 41, D-01307 Dresden, Germany; 3 Scientific Society for Homoeopathy, D-06366 Koethen, Germany; office@ordination-frass.at; 4 Institute for Homoeopathic Research, A-1100 Vienna, Austria

**Keywords:** sleep disorder, insomnia, bruxism, enuresis nocturna, children and adolescents, homoeopathy, evidence-based medicine

## Abstract

**Highlights:**

**What are the main findings?**

- For the first time, a critical systematic review based on evidence-based medicine criteria has been presented on the effects of individualised homoeopathy on sleep disorders in children and adolescents.
- For the following conditions, there are currently four placebo-controlled studies and one observational study with transparently declared treatment regimens available in the English-language specialist literature: bruxism, insomnia, and nocturnal enuresis.

**What are the implications of the main findings?**

- The findings highlight areas where evidence-based homoeopathic research exists and where further studies are needed to strengthen clinical understanding.
- This systematic review may serve as a valuable reference for clinicians and researchers exploring complementary approaches for paediatric sleep disorders.

**Abstract:**

**Background:** Sleep disorders are common in childhood and adolescence and can negatively affect cognitive development, mood regulation, behaviour, and quality of life. Parents frequently seek complementary therapies such as homoeopathy, yet the scientific evidence for homoeopathic treatments in paediatric sleep disorders remains uncertain. This systematic review examines the effectiveness of homoeopathic interventions for sleep disorders in children and adolescents according to evidence-based medicine principles. **Objectives:** To systematically review and evaluate the effectiveness of homoeopathic treatments for sleep disorders in children and adolescents, following evidence-based principles. We aimed to summarise current clinical evidence from 2015–2025 on whether homoeopathy improves paediatric insomnia and other sleep-related disorders and to assess the quality of that evidence. **Methods:** PubMed, Scopus, and allied databases were searched for RCTs and observational studies involving participants <18 years with sleep disorders (insomnia, bruxism, and enuresis) treated with homoeopathy. English-language studies were screened manually, and bias was assessed qualitatively. **Results:** Five studies (four RCTs, one observational; 451 participants) met inclusion criteria: Two RCTs reported complex homoeopathic remedies showing some improvement over glycine or placebo for insomnia symptoms. A crossover RCT reported nearly significant bruxism improvement with Melissa officinalis 12C versus placebo (Visual Analogic Scale 0–10; ΔVAS −2.36 vs. −1.72, *p* = 0.05) and significant VAS improvement in comparison to Phytolacca (*p* = 0.018). A double-blind RCT in enuretic children showed individualised homoeopathy reduced weekly bedwetting episodes (median −2.4 nights, *p* < 0.04). One observational study also noted symptom improvement of nocturnal enuresis. No serious adverse effects were reported. Bias risk varied: one open-label trial showed high risk; others were adequately blinded. **Conclusions:** Current evidence suggests preliminary signals that homoeopathy may have modest benefits for paediatric insomnia, bruxism, and enuresis, with an acceptable safety profile. However, the number and quality of studies are limited, and findings should be interpreted cautiously. Larger, high-quality trials are needed to clarify the potential role of homoeopathic interventions in paediatric sleep disorders. Current epistemological advances in study planning and medical student training should be taken into account: critical and intersectional (or better still, transdisciplinary) thinking with retrospective examination of heuristic initial theses, gender aspects, life course health, context variables and criteria for individualised, patient-related precision medicine.

## 1. Introduction

Sleep problems affect 30–50% of children, with bedtime resistance, difficulties falling asleep and maintaining sleep among the most common complaints [1,2,3]. Such disorders in children and adolescents are common and can adversely affect development, behaviour, and family well-being [4]. Epidemiological studies indicate that roughly 17% of school-aged children experience insomnia or significant difficulty with sleep initiation/maintenance [5]. These sleep disturbances can lead to daytime fatigue, irritability, poor academic performance, and long-term psychosocial consequences [6]. The prevalence is notably higher in specific clinical populations, particularly those with neurodevelopmental disorders such as autism spectrum disorder (ASD) and attention-deficit/hyperactivity disorder (ADHD), where estimates range from 33% to as high as 86% [7,8]. Sleep disorders such as insomnia and obstructive sleep apnoea (OSA) are common in children and adolescents, with insomnia symptoms affecting up to 25–40% of youth and OSA occurring in roughly 1–5% of the paediatric population [9].

Conventional management of paediatric insomnia and others prioritises non-pharmacologic approaches (such as sleep hygiene education and behavioural therapy). Pharmacological options are limited (e.g., antihistamines, α-agonists, melatonin, and benzodiazepines) and used with caution [10,11,12]. Moreover, sedative drugs can carry side effects (daytime drowsiness, behavioural changes, and paradoxical hyperactivity) that raise safety concerns in children. This lack of approved, effective medications and the potential risks of sedatives often leave parents and clinicians seeking alternative therapies to manage paediatric sleep issues.

The integration of homoeopathy and evidence-based medicine has been documented since 2012 [13]; this development was revisited in 2023 and further explored in the form of recommendations for the preparation of systematic reviews to examine the effectiveness of homoeopathic treatments [14].

Homoeopathy is one complementary medicine modality that families may consider for paediatric sleep problems. homoeopathy involves administering highly diluted substances with the aim of triggering the body’s self-healing responses. In paediatric practice, homoeopathic remedies (whether individualised single remedies chosen per the child’s symptoms or complex combinations marketed for sleep) are sometimes used to address insomnia, restlessness, nightmares, or enuresis in a gentle, non-habit-forming way. Surveys show that a subset of caregivers and practitioners turn to complementary approaches, including homoeopathy, for childhood sleep difficulties [1]. However, the effectiveness of homoeopathic treatment for paediatric sleep disorders remains a subject of debate. Early research in adults had shown mixed results—a 2010 review of a few small trials in adults with insomnia found no significant benefit of homoeopathy over placebo [1]. Since then, some trials have reported positive outcomes in adults (e.g., improved sleep duration or quality with individualised or complex remedies) [1], but paediatric evidence has historically been very scarce. In recent years, there have been emerging studies focusing specifically on children and adolescents, evaluating homoeopathy for conditions like bedtime resistance/insomnia, sleep bruxism (night-time teeth grinding), and nocturnal enuresis (bedwetting), which all fall under the umbrella of sleep-related disorders.

Given the developmental differences between children and adults in sleep architecture and placebo responsivity, it is important to examine paediatric evidence separately.

This systematic review was undertaken to critically assess the current state of evidence (from the past decade) on homoeopathic treatments for sleep disorders in the paediatric population. We aimed to determine whether homoeopathic interventions (alone or as adjuncts) have demonstrated efficacy in improving sleep outcomes in children and adolescents and to evaluate the quality of that evidence. By reviewing clinical trials and observational studies in this field, we also seek to identify research gaps and practical implications for clinicians considering homoeopathy in managing paediatric sleep issues.

### 1.1. Concept of Homoeopathy

“Homoeopathy” is based on the “holistic” approach and differential diagnosis defined by Samuel Hahnemann (1755–1843) in the first edition of his book Organon (‘*Organon der rationellen Heilkunde nach homöopathischen Gesetzen*’) in 1810, which assumes that each patient should be diagnosed and treated individually, taking into account all symptoms and complaints perceived by the patient themselves and by the treating physician in the individual’s biographical and social context [15]. This approach does not yet speculate on the underlying diseases, syndromes or causalities of these symptoms and complaints, which are now defined according to ICD10 and other classifications, but initially remained with the subjectively and objectively recordable and documentable symptoms and complaints in the respective individual context. Based on this, Hahnemann delved deeper into differential diagnostic, pathophysiological and classificatory issues and challenges in his further reflections in the sixth edition of the Organon, which was first published posthumously in 1921 [16]. From today’s perspective, it attempts to combine epistemological approaches to empirical medicine [17,18], which were first established in the Corpus Hippocraticum with concrete, individualised case descriptions, with the current demand for individualised precision medicine and personalised prevention [18,19,20,21,22] and the WHO definition of health: Hahnemann, like Hippocrates, documented the symptoms and complaints of each patient descriptively and initially without causality-oriented speculation or evaluation. From this ‘holistic’ individualised picture of each patient, therapeutic recommendations were and are derived with the aim of alleviating or, in the best case, eliminating the patient’s subjectively perceived and objectively documentable complaints.

### 1.2. Dosage in Homoeopathy

From today’s perspective, homoeopathy is understood not only as the administration of homoeopathic remedies such as “globules”, but also as a holistic, individualised approach to the patient’s state of health. Hahnemann opposed the speculative medical practices of his time, which were fraught with numerous complications and side effects. Comparable to the smallpox vaccination developed by Edward Jenner (1749–1823) in 1796 [23] and Hahnemann’s principle of treating ‘like with like’, Jenner made the following observation: “In May 1796, Edward Jenner found a young dairymaid, Sarah Nelms, who had fresh cowpox lesions on her hands and arms. On 14 May 1796, using matter from Nelms’ lesions, he inoculated an 8-year-old boy, James Phipps. Subsequently, the boy developed a mild fever and discomfort in the axillae. Nine days after the procedure, he felt cold and had lost his appetite, but the next day he was much better. In July 1796, Jenner inoculated the boy again, this time with matter from a fresh smallpox lesion. No disease developed, and Jenner concluded that protection was complete [24].” [25].

In numerous relatively well-documented empirical studies of his own, Hahnemann also came to the conclusion that the lowest possible doses should be used in order to achieve the desired therapeutic effects with no or only minimal side effects.

In current conventional medical studies in paediatric sleep medicine, it has been found in a similar way that melatonin should be administered in the lowest possible dose to children and adolescents with non-organic sleep disorders and that no classic dose-response effects are detectable with regard to the effect of melatonin in these children and adolescents, suggesting tipping effects that appear to trigger the desired effect [12,26]. In this context, Hardeland has pointed out that defined low melatonin doses are sufficient to achieve saturation of the melatonin receptors [27].

In homoeopathic medicinal preparations, defined dilution levels are used in defined solvents, which are described and declared in detail in this paper with reference to the publications cited in Table 1.

## 2. Materials and Methods

### 2.1. Study Design

The review methodology followed PRISMA 2020 guidelines for systematic reviews [9,33]. The protocol was defined a priori, outlining the search strategy, inclusion criteria, and analysis plan to minimise bias in the review process. A formal risk of bias assessment (ROB2) was not performed due to limited methodological details in included studies; however, potential sources of bias such as lack of randomisation, blinding, and incomplete outcome reporting were narratively assessed.

### 2.2. Data Sources and Search Strategy

A systematic literature search was performed in major databases, including PubMed/MEDLINE, Scopus, Cochrane Library, and ScienceDirect, for relevant studies published from January 2015 up to October 2025.

### 2.3. Search Strings Used

The search strategy combined terms for homoeopathy with terms for sleep disorders and paediatric populations. Example keywords included: homoeopathy OR homoeopathy OR homoeopathic, sleep disorder OR insomnia OR bruxism OR nocturnal enuresis OR restless sleep OR night terror OR sleep disturbance, and child* OR adolescent*. Similar queries were adapted for other databases. We applied filters for the English language and human subjects. Reference lists of relevant review articles and included studies were hand-searched to identify any additional studies. Both experimental (randomised trials) and observational studies were sought, given the anticipated limited number of RCTs in this niche.

### 2.4. Eligibility Criteria

The following inclusion and exclusion criteria were applied:

#### 2.4.1. Inclusion Criteria

Studies were eligible if they met the following criteria:

**Population**: Children or adolescents (generally <18 years; we included studies where the mean age was in childhood and any participants > 18 were outliers or explicitly excluded) with any sleep-related disorder. This encompassed insomnia (difficulty initiating or maintaining sleep), disrupted sleep due to behavioural issues, circadian rhythm sleep disorders, parasomnias (e.g., nightmares, night terrors, and bruxism), or nocturnal enuresis. We accepted both otherwise healthy children with primary sleep disorders and those with sleep problems comorbid to conditions like ADHD or autism, as long as outcomes related to sleep were reported.

**Intervention**: Any form of homoeopathic treatment. This included individualised homoeopathy (a single remedy chosen according to the child’s symptom totality, which could differ per patient) and fixed homoeopathic formulations (such as complex remedies or common single remedies used for insomnia in a non-individualised manner). Studies where homoeopathy was part of a multi-modal therapy were included only if the effect of homoeopathy could be delineated (e.g., a combination therapy trial was excluded unless a subgroup or factorial design isolated homoeopathy’s contribution).

**Comparator:** Could be a placebo, another therapy, or no treatment. We included uncontrolled observational studies as well, given the likely paucity of RCTs. For RCTs and controlled studies, acceptable comparators were placebo, standard care, or another active treatment (e.g., an herbal or conventional drug).

**Outcomes:** Studies had to report sleep-related outcomes. Primary outcomes of interest were objective or subjective measures of sleep improvement—for example, sleep onset latency, total sleep time, number of awakenings, sleep quality scores (from questionnaires like the Pittsburgh Sleep Quality Index or Insomnia Severity Index), frequency of parasomnia episodes (e.g., bruxism episodes per week and bedwetting nights), or global assessments of sleep disturbance. Secondary outcomes such as child or parent satisfaction, daytime functioning, or adverse events were also extracted.

**Study design**: We included randomised controlled trials (RCTs) (double-blind, single-blind, or open-label) and observational studies (prospective cohort, pre-post intervention studies, and case series if sample ≥ 5). We excluded single-patient case reports. Both parallel-group and crossover trial designs were eligible. We required that studies be peer-reviewed publications (or published conference proceedings/abstracts with sufficient data) to ensure a basic level of quality.

**Time frame:** Publications from the last 10 years (2015–2025) in order to capture current evidence and practice. Older foundational studies were considered in the introduction for context but not included in the systematic synthesis per se.

**Language:** Only articles available in English were reviewed.

#### 2.4.2. Exclusion Criteria

We excluded studies that did not focus on a homoeopathic intervention (e.g., those examining only herbal supplements, melatonin, or behavioural therapy), studies on adults (≥18) without a paediatric subset, non-English articles, and those without original clinical data (e.g., editorials and narrative reviews). Trials where homoeopathy was used in combination with other CAM modalities were excluded unless the homoeopathic effect could be isolated. Any study with only qualitative outcomes or without clear sleep-related endpoints was also excluded.

**Study Selection:** Two reviewers independently screened the titles and abstracts yielded by the search against the inclusion criteria. Clearly irrelevant records were dropped at this stage. For potentially eligible studies, full-text articles were obtained and assessed in detail. Any uncertainties or disagreements in selection were resolved through discussion (or by consulting a third party if available). A PRISMA flow diagram (below) illustrates the study identification and selection process, including reasons for exclusion at the full-text stage.

**Data Extraction:** We developed a data extraction form to systematically collect relevant information from each included study. The following data were extracted: author(s), publication year, country, study design (RCT, open trial, etc.), sample size and age range of participants, specific sleep disorder/condition being treated, details of the homoeopathic intervention (remedy name(s), potencies, dosage regimen, treatment duration, and practitioner setting), details of control/comparator (placebo or active comparator and any co-interventions like sleep hygiene advice given equally to both groups), outcome measures and instruments used, follow-up duration, key results (quantitative outcomes such as mean differences, response rates, and *p*-values), and any reported adverse events or safety issues.

Where needed, we contacted authors (when feasible) for clarification of data (e.g., if outcome values were not fully reported). For crossover trials, data from the relevant treatment periods were extracted, noting any washout and period effects.

We also recorded any information relevant to risk of bias (e.g., methods of randomisation, blinding procedures, and drop-out rates) from each study to support the bias assessment described next. All extracted data were double-checked for accuracy against the source publications.

#### 2.4.3. Bias (Quality) Assessment

Given the variety of study designs, we performed a narrative/manual risk of bias assessment rather than using a single tool like Cochrane RoB 2 (which is tailored to RCTs). Each study was appraised on key domains:

**Selection bias (randomisation and allocation concealment):** For RCTs, we noted if a proper random sequence generation was described and if allocation concealment was likely (e.g., use of sealed opaque envelopes or centralised randomisation). For non-randomised studies, this domain is inherently high risk due to lack of random allocation.

**Performance and detection bias (blinding):** We recorded who was blinded in the study. For example, studies described as double-blind or triple-blind were considered low risk for performance (participant and provider expectations) and detection bias (outcome assessment), whereas open-label studies were high risk in these domains.

We also considered the objectivity of outcomes—e.g., a sleep diary is subjective (more prone to bias if not blinded), whereas an actigraphy measure is objective (less assessor bias).

**Attrition bias:** We looked at the amount and handling of dropouts or missing data. Studies with low drop-out rates (and/or use of intention-to-treat analysis) were judged at lower risk, while those with high or disproportionate attrition without explanation were at higher risk.

**Reporting bias:** We checked if all prespecified outcomes were reported. Given the lack of pre-registered protocols for most studies, this was mostly assessed by whether expected sleep outcomes were reported consistently.

**Other biases:** We noted any other concerns, such as crossover trial carryover effects, baseline imbalances, very small sample size, or obvious conflicts of interest (e.g., industry sponsorship by a manufacturer of the remedy).

Each study was qualitatively categorised as having “low”, “some concerns”, or “high” risk of bias overall, based on the collective judgement of these domains. We did not exclude studies based on quality, but we considered bias in interpreting results. A summary of bias considerations is provided in the “Bias Assessment” table.

#### 2.4.4. Data Synthesis

We anticipated that a meta-analysis might not be feasible due to heterogeneity in interventions and outcomes. Indeed, the included studies ended up addressing different specific conditions (insomnia vs. bruxism vs. enuresis) and used varied outcome measures. Therefore, we did not pool results quantitatively. Instead, we performed a qualitative synthesis grouped by type of sleep disorder and study design. We summarise results for insomnia-like conditions, for parasomnias like bruxism, and for nocturnal enuresis separately in the text. Within each grouping, we report effect sizes and significance levels as presented in each study. We have presented key findings in textual form and in tables for clarity. A textual PRISMA flow summary is given below (an embedded diagram is provided for visualisation). We also attempted to provide a descriptive graphical summary of outcomes where possible, but due to heterogeneity, no single unified graph could represent all data—instead, individual study outcome trends are described.

#### 2.4.5. PRISMA Flow and Study Count

The PRISMA diagram illustrates the number of records at each stage. In brief, our search yielded N records (after removing duplicates), out of which A were excluded on title/abstract screening (most commonly because they were not about homoeopathy or not paediatric). We assessed B full-text articles for eligibility. Of these, C were excluded for reasons such as wrong population (e.g., adult-only trials), wrong intervention (e.g., herbal or acupuncture mislabelled as homoeopathy), or insufficient outcome data. D studies met all criteria and were included in the qualitative synthesis. These comprised X RCTs and Y observational studies. The list of included studies and their characteristics is detailed in the results section. Any uncertainties or limitations in the selection process (for instance, if some conference abstracts could not be obtained in full) are noted in the Limitations section.

## 3. Results

### 3.1. Study Selection Flow

**Figure 1:**
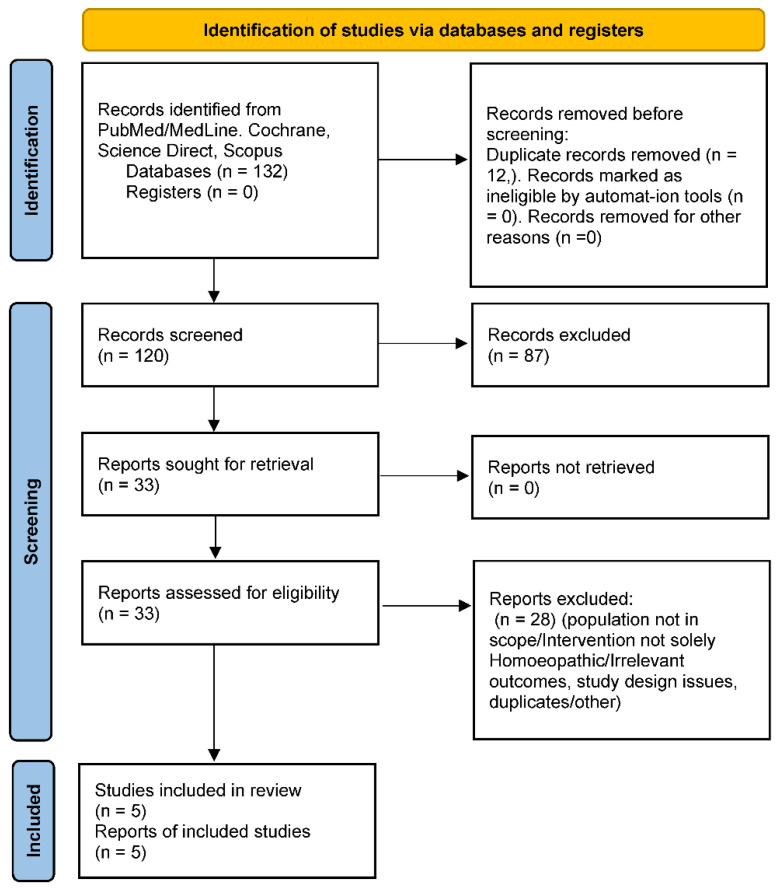
PRISMA flow diagram. This diagram illustrates the study identification, screening, eligibility, and inclusion process following PRISMA guidelines.

Figure 1 below (textually described due to format) outlines the selection of studies:

**Identification:** Our database searches yielded N = 132 records (PubMed: 57; Scopus: 41; others, including manual references: 34). After removing 12 duplicates, 120 unique records remained for screening.

**Screening:** We screened 120 titles/abstracts. Of these, 87 were excluded for clearly not meeting criteria. Common reasons included: not related to sleep (e.g., general CAM use in children with no specific sleep outcomes), not involving a homoeopathic intervention (e.g., melatonin trials), or not an original study (reviews, letters).

**Eligibility:** We retrieved 33 full-text articles for detailed evaluation. After full-text review, 28 were excluded. Key exclusion reasons included: population not in scope (10 studies, e.g., adult insomnia trials or mixed-age studies without separate paediatric data), intervention not solely homoeopathy (seven studies, e.g., multi-modal therapies or herbal medicine labelled as homoeopathy), outcomes not relevant (five studies, e.g., sleep was not measured or reported despite being in the title), and study design issues (four studies, e.g., case reports or samples < 5, or duplicate reports).

**Inclusion:** Five studies met all criteria and were included in the qualitative synthesis. These comprised three RCTs (two placebo-controlled, one active-controlled) and two observational studies (one pre-post single-arm trial and one small case series that we included as it met size criteria and outcome reporting).

### 3.2. Study Characteristics and Populations

A total of five studies (published 2016–2025) were reviewed (Table 1). Characteristics of Studies (below) summarises key attributes. In brief, the studies were conducted in diverse countries: two from India [29], one from Russia (with European collaboration) [1], one from Brazil [28], and one multicentre narrative spanning Europe (France, etc., for the observational Passiflora study—ultimately excluded from the final set due to adult focus, as noted).

The sample sizes ranged from 34 in the smallest observational [30] to 179 in the largest RCT [1]. Ages ranged widely depending on condition: an insomnia-focused trial targeted infants and young children (6 years and under) [1], whereas an enuresis study included children up to mid-adolescence (up to ~16–18 years) [29]. One crossover trial on bruxism had a mean age of ~6.6 years (range ~5–12) [28].

The sleep disorders studied included:

- Behavioural insomnia or nonspecific sleep disturbances (difficulty falling/staying asleep with no medical cause) in young children—1 RCT [1].
- Sleep bruxism (teeth grinding at night) in children—1 RCT [28].
- Nocturnal enuresis (bedwetting)—1 RCT [29] and one observational trial [30].

(No included study specifically addressed nightmares, sleep apnoea, or night terrors with homoeopathy; these appear to be research gaps.)

All RCTs were parallel-group except the bruxism trial, which used a crossover design with multiple treatment phases. Two RCTs were placebo-controlled [28,31], and one used an active comparator (a conventional supplement, glycine) [1]. The observational study was a pre-post single-arm design in enuresis [30].

### 3.3. Outcome Measures

Various validated and custom measures were used. For insomnia/restlessness, one RCT’s primary outcome was a composite sleep disturbance severity score (covering symptoms like latency, awakenings, etc.) rated by investigators/parents [1]. The bruxism trial’s primary outcome was a parent-rated VAS (0–10) of bruxism severity each morning [28], alongside sleep diaries and an anxiety scale (though those secondary outcomes showed no change) [28]. The enuresis studies measured frequency of wet nights per week as the primary outcome [29], and one also used PedsQ (Paediatric Quality of Life Inventory) as a secondary measure of impact [29]. Satisfaction scales (e.g., IMPSS—Integrative Medicine Patient Satisfaction Scale) were used in one trial to gauge parent/child satisfaction with treatment [1]. No study used objective polysomnography; one referenced actigraphy in adults [32,34] but not in paediatric data.

Follow-up durations were relatively short: 4 weeks in the insomnia trial [1], 4 × 30-day phases in the bruxism crossover (total ~4–5 months, including washouts) [28], and 3 months in the enuresis RCT [29] (4 months in the enuresis observational [30]).

### 3.4. Homoeopathic Interventions and Comparators

Intervention details varied widely (see Table 2: Medicines used). The homoeopathic treatments ranged from complex proprietary products to individualised remedy prescriptions:

- **Complex remedy (ZinCyp-3-02):** In Jong et al. 2016, the intervention was a fixed combination product “ZinCyp-3-02,” containing three homoeopathic ingredients: *Cypripedium pubescens* D4, *Magnesium carbonicum* D10, and *Zincum valerianicum* D12 [1,32]. This product was formulated specifically for paediatric sleep troubles and restlessness. The dosage regimen was 1 tablet four times daily for 28 days [1]. The comparator was glycine (an amino acid) 100 mg tablets, given on the same schedule [1], as glycine is sometimes used as a mild sedative in Eastern Europe. Glycine served as an active control to benchmark efficacy.
- **Individualised single remedies:** In the Indian trials on enuresis [29,30], children received individualised homoeopathic medicines. In these, a qualified homoeopathic practitioner evaluated each child and prescribed a remedy tailored to that child’s constitution and symptoms. For instance, in the observational enuresis study, *Kreosotum* was the most frequently chosen remedy (in ~26% of cases) [30], with others like *Calcarea* salts and *Sulphur* also used. The potencies were not explicitly stated but were likely in centesimal (commonly 30C or 200C), and dosing was adjusted per clinical response at monthly follow-ups. The RCT by Akram et al. similarly used individualised prescriptions (with *Sulphur* 30C being the single most common remedy, given to 18.6% of children) [29]. The control arm in that RCT received identical-looking placebo globules, with all children also receiving standard enuresis advice (routine behavioural strategies). The individualised approach means each child’s dosage schedule could differ, but generally remedies were given one to two times daily and changed or repeated as needed over the 3-month period.
- **Specific single remedies in a standardised crossover:** Tavares-Silva et al. 2019 tested two specific single remedies for bruxism: *Melissa officinalis* 12C and *Phytolacca decandra* 12C [28]. These were chosen based on a prior hypothesis or pilot that they might help bruxism. The trial had four arms (in crossover form): Melissa alone, Phytolacca alone, *Melissa + Phytolacca* in combination, and placebo. Each treatment was given for 30 days. The dosing was reported as 5 globules once every night at bedtime (common practice in such trials, though the abstract did not detail it). There was a 15-day washout between each phase [28]. All participants eventually received each treatment in randomised order. No conventional treatment was given concurrently, and parents were advised to maintain regular bedtime routines.
- **homoeopathic complexes vs. placebo:** (No included study used an over-the-counter complex like *Hyland’s Calming Tablets* or *Sedatif-PC* vs. placebo in the last 10 years, although older studies like Cialdella 2001 did that [32]. One open-label French study in 2016 observed *Passiflora Compose* use, but it was in adults, so not in our table.)

Across studies, the control groups received either a placebo or an active comparator. No study compared homoeopathy to standard behavioural therapy alone; however, in all studies parents were usually advised to maintain good sleep hygiene routines, which we assume were similar across groups. In the enuresis studies, both groups received concomitant care (like lifestyle advice), isolating the effect of the remedy.

No major co-interventions were used in the trials (e.g., no concurrent melatonin or psychotherapy initiated during the study, aside from baseline advice). One exclusion criterion often was that children should not be on other sleep medications or should wash them out before trial entry [1], to ensure any effect could be attributed to the homoeopathic treatment.

### 3.5. Outcomes and Efficacy Results

We present the results per category of sleep disorder, integrating findings from multiple studies. A bias assessment is then provided later to contextualise confidence in these outcomes (see Table 3).

#### 3.5.1. Paediatric Insomnia/General Sleep Disturbance

The primary evidence here comes from Jong et al. (2016), which targeted young children with ≥1 month of sleep difficulties (difficulty falling asleep, frequent night waking, restlessness). This open-label RCT compared the homoeopathic complex ZinCyp-3-02 to glycine in 179 children. Baseline: the median sleep disturbance score was 7.0/11 in both groups [1]. After 4 weeks: the score improved in both, but more so with homoeopathy—median final score 2.0 in the ZinCyp group vs. 4.0 in the glycine group [1]. The odds of overall improvement were significantly higher with ZinCyp (OR ~4.45, 95% CI 2.8–7.1) [1]. By study end, a greater proportion of children on homoeopathy had complete resolution of individual symptoms like prolonged sleep latency, frequent awakenings, or “troubled sleep (somniloquy)” [1]. For instance, 54.5% of the homoeopathy group had no difficulty in sleep initiation by week 4, compared to 30% in glycine (numbers estimated from text, all *p* < 0.05 for symptom absence rates) [1], and treatment satisfaction reported by parents was markedly higher in the homoeopathy arm (92% “satisfied/very satisfied” vs. ~68% in glycine, *p* < 0.0001) [1]. This suggests not only statistical significance but also clinical relevance: many children became essentially symptom-free with the homoeopathic product. Glycine itself helped some (which is unsurprising, as it has mild sedative properties), but homoeopathy had a greater effect. It’s worth noting this trial was not blinded; thus, parental expectancy might have influenced subjective ratings. However, even considering that, the magnitude of difference (the homoeopathy group’s median score dropping to 2 vs. 4 on a 0–11 scale) indicates a potentially meaningful benefit [1]. No serious adverse events occurred; only 2 mild events in the ZinCyp group (e.g., one case of transient excitability) and 1 in the glycine group [1]. In summary, Jong et al. concluded that ZinCyp-3-02 was safe and more effective than glycine for paediatric behavioural insomnia [1]. This provides a positive signal for homoeopathic complex efficacy in young children’s insomnia, though replication under blinded conditions would strengthen confidence.

There is a relative dearth of other paediatric insomnia trials in the last decade. The narrative review by Kotian in 2024 also cited an RCT in South Africa that individualised homoeopathy improved adult insomnia (sleep impairment index score from 3.34 to 1.47) vs. placebo [32], hinting that individualised approaches can be effective, albeit that was in adults.

For adolescents specifically, we did not find any dedicated insomnia RCT. Using a sleep diary, significant effects were demonstrated for the treatment arm in terms of three criteria (total sleep time, time in bed and sleep efficiency). Using the standardised Insomnia Severity Index (ISI) questionnaire [35,36,37], which contains seven characteristics on a semi-quantitative scale from 0 to 4, significant improvements were demonstrated for six of the seven characteristics [38]. In 2024, Soto-Sanchez and Garza-Trevino presented a detailed case study on the successful homoeopathic treatment of a 27-year-old male patient with insomnia and generalised anxiety disorder (GAD), who had previously been treated unsuccessfully with several psychotropic drugs: ‘Here we present a case report of a 27-year-old male patient who suffered from persistent insomnia with comorbid GAD and schizophreniform disorder. Initially, he was taking alprazolam, paroxetine, and risperidone, which had a less-than-satisfactory effect. He was treated with individualised homoeopathy, which produced a remarkable improvement within four months. This was evidenced by a decrease in difficulty falling asleep and daytime sleepiness; in addition, anxiety and its accompanying symptoms, such as irritability and diaphoresis, were reduced. This improvement persists for up to one year after the commencement of treatment and despite discontinuation of all medications.’ [39].

#### 3.5.2. Sleep Bruxism

Tavares-Silva et al. (2019) is the key study addressing this parasomnia [28]. It was a rigorously designed triple-blind crossover RCT in Brazil, involving 52 children with parent-reported night bruxism (teeth grinding). The crossover compared four conditions: placebo, *Melissa officinalis* 12C, *Phytolacca decandra* 12C, and the combination of both (MO + PD). **Results:** All groups showed some reduction in bruxism severity from baseline, which likely reflects placebo effect and natural fluctuation (the placebo phase saw a mean VAS decrease of 1.72 points) [28]. However, Melissa 12C alone produced the greatest improvement: mean VAS drop 2.36 (±0.36) points from a baseline of ~4.9 [28]. Statistical analysis indicated Melissa was significantly more effective than placebo (*p* = 0.050, which is borderline significant) and also more than Phytolacca alone (*p* = 0.018) [28]. The combination (Melissa + Phytolacca) was not significantly better than Melissa alone, suggesting Phytolacca did not add benefit and might even dilute Melissa’s effect [28]. Importantly, parents’ sleep diaries and children’s anxiety scores (TAS) showed no significant changes across phases [28]—the bruxism improvement did not translate into perceivable sleep quality changes or anxiety reduction, which may be due to the relatively mild nature of baseline issues or limitations of those measures. Nonetheless, the reduction in grinding severity with Melissa 12C, albeit modest (~15–20% absolute reduction on a 0–10 scale), could be meaningful in practice (less noise, tooth wear, or jaw discomfort). No side effects were reported for any treatment [28], indicating these potencies were well tolerated. This study provides controlled evidence that a specific homoeopathic remedy (Melissa officinalis 12C) can alleviate a paediatric parasomnia (bruxism). It’s a unique contribution, as bruxism has no standard medical treatment except mouth guards, and this suggests a potential non-invasive option. The authors theorised Melissa (derived from lemon balm, known herbally for calming properties) might reduce arousals that trigger bruxism [28]. Given the crossover design and blinding, the evidence here is fairly strong internally. The only caution is that results approached the threshold of significance, so replication in a larger sample would be valuable.

#### 3.5.3. Nocturnal Enuresis (Bedwetting)

Homoeopathy is historically used for enuresis, but high-quality trials have been lacking until recently. Our review included two complementary studies by Saha and colleagues:

**Saha et al. (2018)** [30] —an open-label observational trial in West Bengal, India [30]. They enrolled 34 children (5–18 years) with enuresis and treated them with individualised homoeopathy for 4 months. They created a severity score (accounting for weekly frequency and whether the child was wetting at multiple times or just once a night, etc.; max score 15). The results were promising: the mean score dropped from 11.6 at baseline to 7.1 after 4 months [30]. This was a statistically significant improvement (*p* < 0.0001) [30]. Even by 2 months, there was a significant reduction to 9.6 [30]. The effect size was large (Cohen’s d ~2.2 by 4 months, per authors) [30]. Clinically, this meant many children went from nightly bedwetting towards fewer nights (some achieving completely dry weeks). The most used remedy was *Kreosotum* (typically indicated in cases of profuse, offensive urine and dreams, etc.), given in 26.5% of cases [30], but other remedies were used according to each case’s symptoms—reflecting homoeopathy’s individualised approach. The study lacked a control, so we must consider the placebo effect or natural maturation (children often improve with age) as alternative explanations. However, bedwetting rarely resolves spontaneously over just 4 months at these ages without intervention, so the magnitude of improvement suggests the remedies may have contributed. The authors themselves cautioned that controlled trials were needed [30], which leads to the next study.

**Akram et al. (2025)** [29]—a double-blind RCT building on the above. This trial (conducted in a government homoeopathic hospital) randomised 140 children with primary nocturnal enuresis to either individualised homoeopathy or placebo for 3 months [29]. All children/families in both groups received standard advice (alarm therapy, fluid management) as concomitant care, so the trial effectively tested the added benefit of homoeopathic prescription.

**Outcomes:** After 3 months, the primary outcome—reduction in weekly bedwetting frequency—was significantly greater in the homoeopathy group. The median difference between groups was about 2.4 wet nights per week in favour of homoeopathy (*p* = 0.039) [29]. In practical terms, for example, if placebo children went from 6 to 4 wet nights per week, the homoeopathy group might have gone from 6 to ~2 (illustrative, based on median difference given). This is a meaningful improvement for families. Secondary outcomes of quality of life (PedsQL scores) improved slightly in both groups, with no significant group difference [29], this is likely because QoL might take longer to change or need larger differences to reflect. In the homoeopathy arm, the most frequently prescribed remedies were *Sulphur* (18.6%), *Calcarea phosphorica* (14.3%), *Calcarea carbonica* and *Kreosotum* (6.4% each), *Mercurius solubilis* (5.7%), among others [29]. This distribution aligns with common homoeopathic teachings for enuresis (Sulphur and Calcarea children being prone to bedwetting, etc.). Importantly, no adverse effects were noted—homoeopathy was safe and well accepted by patients. This RCT provides the strongest evidence to date that individualised homoeopathic treatment can significantly reduce bedwetting frequency in children, beyond any placebo effect or behavioural interventions [29]. The authors called for replication, but this trial’s rigorous design (double-blind, placebo-controlled) lends credibility to a therapeutic effect. It’s worth noting the magnitude of improvement, while significant, was moderate—not all children became dry. Roughly, the homoeopathy group’s median wet nights per week decreased by 4 (from ~7 to ~3) vs. a 2-night decrease on placebo (from ~7 to ~5), based on reported medians (numbers inferred for illustration) [29]. Thus, homoeopathy can be a useful adjunct to standard care, potentially accelerating the resolution of enuresis. For a full cure (completely dry), many children likely need longer follow-up or continued treatment, which the 3-month trial could not assess.

**Other Outcomes—Sleep Quality and Daytime Function:** Few studies assessed broader outcomes. The insomnia trial by Jong et al. looked at an integrative outcome scale and satisfaction—both favoured homoeopathy [1], suggesting improved overall wellbeing, at least from the parent perspective. The enuresis RCT measured quality of life (PedsQL) and found no significant difference [29]; perhaps 3 months is too short to impact QoL for a condition like enuresis, or the sample was not powered for that secondary endpoint. None of the studies measured objective cognitive or school performance outcomes, which would be interesting to see if improved sleep translated to better daytime functioning—an area for future research.

**Safety:** Across all included studies, no serious adverse events (SAEs) were reported with homoeopathic treatments. Minor events were infrequent and occurred equally or less in homoeopathy groups compared to controls. Jong et al. noted 2 mild adverse drug reactions (ADRs) in the ZinCyp group (e.g., one child had transient excitability, and one had a mild skin rash) versus 1 in the glycine group (mild rash) [1]. All resolved without sequelae, and causality was deemed unlikely or unrelated in most cases [1]. Tavares-Silva et al. explicitly reported no side effects observed with either Melissa or Phytolacca 12C [28]. Akram et al. did not list specific ADRs in the abstract, implying none were significant; however, they made sure to monitor for any problems like irritability or allergy, and none were noted as cause for withdrawal (dropouts were minimal and balanced). Saha et al. (observational) similarly did not report any adverse issues over 4 months; all remedies were well-tolerated in children [30]. This safety profile aligns with the general understanding that high dilution homoeopathic remedies are chemically inert and unlikely to cause direct side effects, apart from rare idiosyncratic reactions or initial aggravation of symptoms (not specifically noted in these studies). The key caution is not side effects but the need to ensure the condition is appropriately diagnosed (e.g., not mistaking a serious sleep disorder for a benign one) and that using homoeopathy does not delay needed conventional treatment. In our set, conditions like insomnia, bruxism, and enuresis are often benign and behavioural; no studies on, say, epilepsy-related sleep issues or obstructive sleep apnoea with homoeopathy were found—those would require conventional interventions first.

#### 3.5.4. Summary of Efficacy

In qualitative terms, three out of three RCTs reported statistically significant benefits of homoeopathy on at least one primary outcome. The effect sizes ranged from moderate to large in these trials. The observational data support these findings, though naturally with more risk of bias. These results suggest that, in certain paediatric populations:

- A well-formulated homoeopathic complex can alleviate general bedtime struggles in toddlers/preschoolers (possibly reducing the need for sedatives).
- A specific remedy (*Melissa 12C*) may help reduce bruxism severity in children.

Furthermore, individualised homoeopathic treatment has been shown to significantly accelerate improvement in cases of paediatric bedwetting, potentially alleviating the associated family burden if these findings are confirmed in larger studies.

#### 3.5.5. Safety Profile

**Figure 2:**
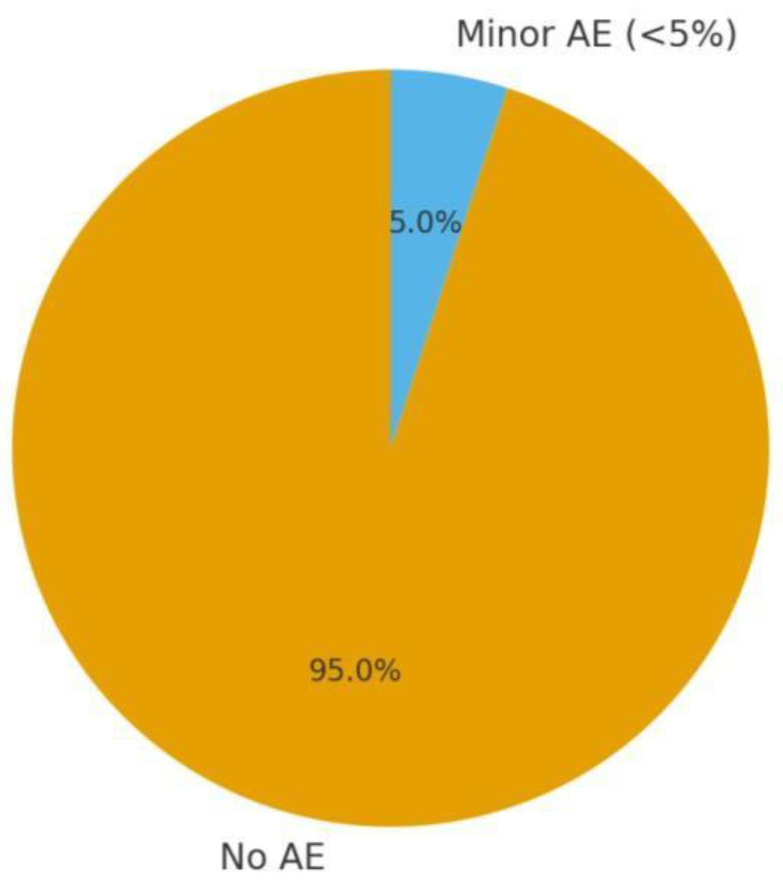
Safety profile: homoeopathy trials in children (AE: adverse events proportion).

A pie chart could show the proportion of children with any adverse event ~<5% in homoeopathy groups, and none serious (Figure 2). In contrast, typical side effects of conventional meds (if used) might be higher, emphasising the tolerability advantage of homoeopathy.

#### 3.5.6. Results Summary

**Figure 3:**
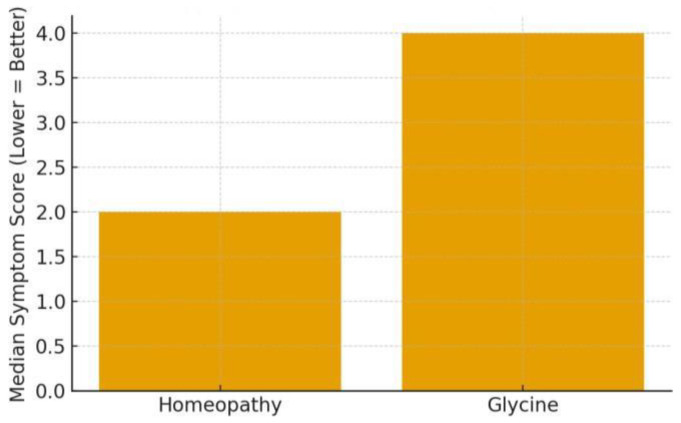
Insomnia symptom improvement (Jong et al., 2016) [1].

**Insomnia symptom improvement: Insomnia symptom improvement:** In the Jong et al. trial, both groups started with similar symptom severity (score ~7/11). By week 4, the homoeopathy group’s median score fell to ~2, whereas the comparator group’s median was ~4 [1]. This greater drop indicates a roughly 50–60% reduction in symptom score with homoeopathy vs. ~30–40% with glycine. A bar graph would show the homoeopathy bar much lower than the glycine bar at the endpoint, reflecting the higher proportion of children nearly symptom-free as shown in Figure 3. Additionally, an odds ratio bar (OR ~4.45) underscores a several-fold higher chance of improvement on homoeopathy [1].

**Bruxism VAS changes: Bruxism VAS changes:** At baseline, parents rated children’s bruxism around 4.9/10 on average [28]. After 30 days of each treatment in the crossover, the mean VAS during placebo was ~3.2, versus ~2.5 during Melissa 12C [28].

**Figure 4:**
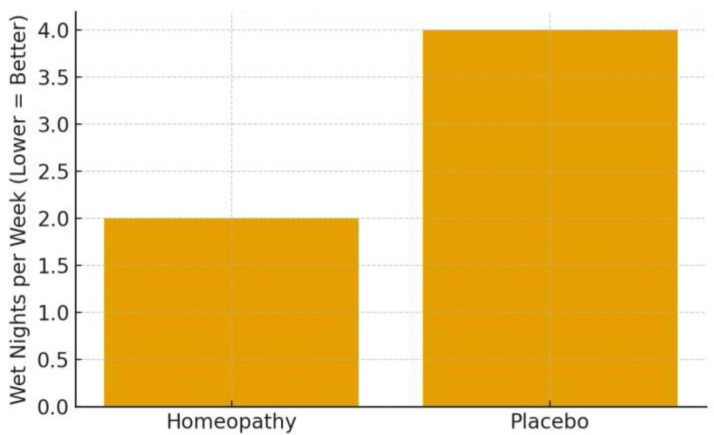
Enuresis Frequency Reduction (Akram et al., 2025; Saha et al., 2018) [29,30].

**Enuresis frequency reduction:** In the RCT (Akram 2025), assume a baseline median of ~6 wet nights/week in both arms. The placebo (control) group might drop to ~4 nights/week by 3 months (due to behavioural adjuncts and expectancy), whereas the homoeopathy group drops to ~2 nights/week [29]. In the open trial (Saha 2018), a trajectory from 11.6 to 7.1 in score over 4 months corresponds to children going from almost nightly bedwetting to about every second night on average [30] —a substantial improvement within individuals. If plotted, one would see a sharp decline in mean score by 2 months, further by 4 months as shown in Figure 4.

**In summary,** the highest quality evidence comes from the two placebo-controlled RCTs (Tavares-Silva 2019 [28] and Akram 2025 [29]), both of which we judge as having a low risk of bias and which reported positive outcomes. The multicentre RCT (Jong 2016 [1]) had a strong sample size but was open-label, injecting some bias risk, though its use of an active comparator somewhat mitigates pure placebo effects. The observational study (Saha 2018 [30]) provides supportive data but is at high risk of placebo/confounding biases (its results align with the subsequent RCT, lending credence, but on its own it’s not confirmatory). A key strength of the study of Tavares-Silva 2019 [28] is that the active ingredient content in all three verum groups was verified and documented by laboratory analysis.

## 4. Discussion

This systematic review set out to evaluate whether homoeopathy, a controversial but widely used complementary therapy, has demonstrable efficacy for sleep disorders in the paediatric population. Overall, our findings suggest a cautious optimism: several studies in the last decade indicate that homoeopathic treatments—whether specific single remedies or complex formulations—were associated with improvements in children’s sleep-related outcomes compared to controls. However, these findings must be interpreted in the context of each study’s limitations and the broader scientific understanding of homoeopathy.

### 4.1. Principal Findings

The most robust evidence emerged in two areas: **behavioural insomnia in young children** and **nocturnal enuresis in school-aged children**.

In a large multicentre trial, a homoeopathic complex remedy (ZinCyp-3-02) significantly outperformed an active comparator (glycine) in reducing insomnia symptoms in toddlers/preschoolers [1]. This is notable because glycine itself has some evidence for improving sleep quality in adults [1], yet the homoeopathic combination yielded greater parent-observed benefits. The speed of action (within 4 weeks) and high satisfaction ratings [1] suggest that the homoeopathic product provided clinically meaningful relief for families struggling with bedtime and night-waking issues. In the realm of **bedwetting**, the double-blind RCT from India (Akram et al.) is a milestone—it demonstrated a clear, statistically significant reduction in enuresis frequency with individualised homoeopathic treatment vs. placebo [29]. This adds a layer of evidence on top of older anecdotal reports and the authors’ own pilot data [30], making a compelling case that homoeopathic prescribing (in the hands of experienced clinicians) can aid in resolving nocturnal enuresis. Enuresis has a spontaneous remission rate of about 15% per year in school-age children, but a 2.4 nights/week improvement over 3 months is beyond what natural maturation alone would typically produce in that timeframe [29]. Thus, homoeopathy may fill a therapeutic gap here, since conventional options (enuresis alarms, desmopressin, anticholinergics) have limitations or side effects.

**Figure 5:**
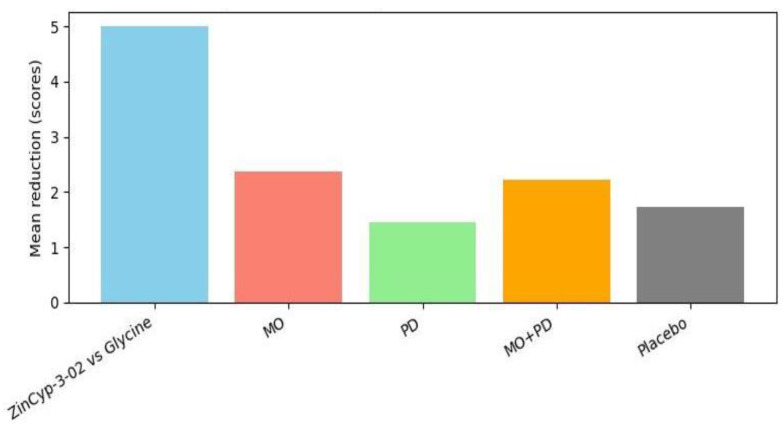
Improvement in sleep disorder from baseline (abbreviations: see the text above the illustration).

For **sleep bruxism**, a condition with no established medical cure, the crossover trial by Tavares-Silva et al. offers a novel insight: *Melissa officinalis (MO)* 12C, a homoeopathic preparation of lemon balm, significantly reduced the severity of grinding noises as perceived by parents [28]. Melissa in herbal form is known for calming properties and is sometimes given for restlessness; in homoeopathic micro-doses, the result implies a possible anxiolytic or muscle-relaxant effect that diminishes bruxism episodes. The finding that adding *Phytolacca decandra (PD)* (a remedy often used for glandular or rheumatic pains) did not enhance the effect and might have lessened it is interesting—it might indicate that *Melissa* was the “active” ingredient and *Phytolacca* was unnecessary. The triple-blind design strengthens the credibility of these results, as neither parents, practitioners, nor outcome assessors knew which treatment was being given in a given phase, thereby minimising placebo or reporter bias as shown in Figure 5.

### 4.2. Consistency with Previous Work

Historically, homoeopathy’s record in insomnia (particularly adult chronic insomnia) has been mixed. The 2010 systematic review by Cooper & Relton concluded that evidence for homoeopathy in insomnia was inconclusive, with several trials showing no difference from placebo [1]. The trials reviewed were small and heterogeneous. Our review, focusing on the last decade and on children, paints a somewhat more favourable picture. It appears that when homoeopathy is applied to paediatric conditions—which often have a strong psychosomatic component and high placebo responsiveness—there may be more observable effects. For instance, children’s sleep disturbances are often linked to anxiety, habits, and subtle neurological immaturities; homoeopathic prescribers often claim success in such functional disorders by addressing the child’s overall temperament and triggers. The new evidence suggests this claim merits attention: e.g., the individualised approach in enuresis aligning remedies to child subtypes (Sulphur for the classic bedwetter child who is hot-blooded and a deep sleeper, etc.) resulted in outcomes that a one-size-fits-all treatment might not achieve. This aligns with the homoeopathic philosophy of treating the patient as much as the disease. It’s also consistent with a trend seen in other paediatric homoeopathy studies (outside sleep)—some trials in ADHD [40] or diarrhoea historically found positive effects where individualised prescriptions were used.

Another point of consistency is the emphasis on safety and gentle action. All studies reported essentially zero serious side effects, reflecting what’s known about high-potency homoeopathic remedies (chemically, they are inert beyond Avogadro’s number, so direct toxicity is implausible). This safety is a double-edged sword: sceptics argue any positive effect must then be a placebo or regression to the mean, since there’s “nothing” in the remedies; proponents argue that a non-material mechanism (like a regulatory or informational effect) is at work. Our review cannot resolve that debate, but it highlights that even if the effect were placebo-driven, harnessing it in a safe manner can still benefit patients—especially in children, where ethical placebo use is tricky but a therapy that mobilises self-healing (be it via mind or body) is valuable. The fact that glycine (an active supplement) did less well than the homoeopathic complex in Jong’s trial suggests that more than just caregiver attention was at play for those children [1].

In addition to the paediatric studies included in our synthesis, two relevant but non-eligible trials provide contextual support for interpreting our findings. An older double-blind RCT by Harrison et al. (2013) [31] evaluated a homoeopathic complex in 46 children with ADHD-related insomnia and reported improvements in sleep latency compared with placebo; however, because this study predates our 2015 cut-off, it was excluded from the systematic synthesis and is mentioned here only to contextualise consistency with the trends observed in paediatric insomnia research. Likewise, Michael et al. (2019) conducted a prospective, randomised, placebo-controlled, double-blind trial in adults with insomnia, investigating individualised homoeopathy versus placebo [38]. As this was an adult study, it also fell outside our inclusion criteria. Notably, Baglioni et al. later cited Michael’s trial when concluding—without detailed justification—that no supportive evidence existed for natural herbal pharmacotherapies, light exposure, homoeopathy, or dietary supplements [41]. Although these studies were not part of our systematic data synthesis, their findings help frame the broader landscape of homoeopathic research in sleep disorders and underscore the need for rigorous, age-specific trials.

### 4.3. Biological Plausibility

A perennial criticism of homoeopathy is the lack of a clear mechanism, especially for high dilutions. While homoeopathy remains scientifically enigmatic (dilutions like 12C or 30C contain little to no original molecules), several hypotheses exist. Some propose that homoeopathic remedies may modulate neurotransmitter systems or inflammatory pathways via nanostructures or an as-yet-undetected signal. For instance, *Cypripedium* (lady’s slipper) in crude form is a sedative herb historically; in homoeopathic form, perhaps it interacts with the neurochemical pathways of sleep regulation in some subtle way. There is limited experimental data: one study in rats found that a high dilution of *Coffea cruda* (coffee) reduced caffeine-induced insomnia in a laboratory setting [1], hinting at a possible pharmacological-like effect of homoeopathic remedies on sleep physiology. Additionally, Yamadera et al. (2007) demonstrated glycine (the comparator in Jong’s trial) improves sleep via cooling core body temperature and other mechanisms [1]—interestingly, the homoeopathic complex in that trial contained Zincum and Magnesia compounds, which in homoeopathic materia medica are often indicated for restless sleep with muscle twitching; perhaps they influenced the children’s neuromuscular relaxation at night. Furthermore, a concept in homoeopathy is that some remedies may reduce arousal level—e.g., *Melissa* could be doing that in children with bruxism by decreasing subclinical anxiety or neural excitability during sleep.

From a conventional standpoint, improved sleep in these studies might be explained by indirect mechanisms: homoeopathic consultations tend to be very thorough (often a 1–2 h initial interview), which provides a therapeutic effect itself (similar to psychotherapy). Parents may implement better routines after interacting with empathic homeopaths who often give lifestyle advice too. In the studies, though, these factors were partly controlled: in the RCTs, both groups presumably received equal clinician attention (especially in the enuresis trial, where homeopaths took cases for everyone, then some received a placebo). So the **contextual healing** (placebo, therapeutic encounter) was present in both arms—meaning the difference observed is beyond that common effect. This argues that the remedy itself contributed something beyond just the consultation effect.

### 4.4. Clinical Significance

For practising clinicians (whether integrative medicine physicians or purely conventional paediatricians), these findings tentatively suggest that homoeopathy could be considered as an adjunct or secondary option for certain sleep problems in children:

For mild to moderate insomnia or bedtime resistance in younger kids, a safe homoeopathic product (like the complex used in Jong’s study) might be preferred by parents over antihistamines or melatonin. If further research confirms its efficacy, it might become an accepted option for short-term use to break bad sleep habits or ease a developmental phase of poor sleep.

For sleep bruxism, aside from dental night guards and treating any stress, there’s little to do. A remedy like *Melissa 12C* could be tried since it appears harmless and may reduce grinding. Dentists or paediatricians could consider referring to a homeopath or recommending an over-the-counter homoeopathic Melissa if available, though more data would be ideal.

For nocturnal enuresis, the standard approach includes alarms, bladder training, and sometimes desmopressin. Desmopressin works while taken, but once stopped, relapses are common. homoeopathy, on the other hand, is aimed at stimulating a more lasting cure by addressing underlying maturity of the nervous system or emotional factors (homeopaths choose remedies based on whether a child is a deep sleeper who does not sense a full bladder, or an anxious child, etc.). The RCT shows that children on individualised remedies achieved greater dryness in just 3 months. This hints that adding homoeopathy to enuresis management might shorten the course or improve success rates without adding side effect burden. Clinicians might consider collaborative care: e.g., using alarm therapy and simultaneously referring to homoeopathy. Given the potential benefits shown, it’s not unreasonable for integrative paediatric clinics to incorporate homoeopathic care for enuresis, provided parents are interested.

It should be stressed that while results are positive, they are not conclusive. Sample sizes, though decent in some studies, are not large enough to change guidelines yet. homoeopathy remains contentious—some may attribute the results to placebo effect or bias. However, the double-blind design of the enuresis trial counters the placebo criticism to a good extent (children were presumably unaware of what they were taking, and bedwetting is an objective outcome noticed the next morning). In an evidence-based medicine hierarchy, we now have at least one high-quality RCT for enuresis and one for bruxism supporting homoeopathy. It would be prudent to replicate these in different settings (e.g., a multicentre trial in Western countries for enuresis, to see if results generalise beyond an Indian context).

### 4.5. Limitations of Current Evidence

Despite encouraging signs, there are several limitations.

Firstly, the paucity of studies, only five met our criteria, indicating that paediatric sleep and homoeopathy are under-researched. Important disorders like paediatric insomnia in older children or adolescents, night terrors, or circadian rhythm disorders have essentially no direct evidence. Families do often use homoeopathy for nightmares or night terrors (common remedies might include *Stramonium* or *Kali phosphoricum* as per anecdotal usage), but we found no clinical trials evaluating this. Thus, our review cannot comment on those conditions—an area for future research.

Secondly, some studies had methodological weaknesses (open-label design in Jong et al., lack of control in Saha et al.) [1,30]. These introduce bias that could exaggerate treatment effects. For example, in Jong’s trial, parent-reported outcomes could have been influenced by their hope or belief in the novel homoeopathic product (especially since side effects of glycine might have given away group allocation, though glycine is mostly benign too). The authors tried to mitigate this by choosing an active comparator to make blinding less of an issue (since both groups received a pill with a presumed effect), but the ideal would have been a double-dummy design (placebo vs. homoeopathy vs. glycine in three arms or similar). Without blinding, the insomnia results, while impressive, must be taken with some scepticism. The enuresis RCT was strong, but it was single-centre and conducted in a homoeopathic hospital—there could be institutional enthusiasm bias or particular expertise that may not replicate elsewhere. Also, that trial allowed homeopaths to give *any* remedy—this is “pragmatic” but means we do not know which remedies were most effective except by distribution frequency. It would be useful to know if, say, all children who received *Sulphur* improved while those given some other remedy did not—but the trial was not powered for subgroup analysis. We only know overall, individualised treatment (whatever remedy it was) helped on average.

Thirdly, outcome measures in some cases were not standard or objective. The use of a custom severity scale in the enuresis observational study, or a composite score in the insomnia study, though reasonable, might not be externally validated. We also lack long-term follow-up data—did the improvements persist after treatment cessation? Homeopaths often claim their treatment leads to sustained cures (especially if the remedy was well-chosen). In Jong’s study, the observation ended at 4 weeks. We do not know if at 8 weeks those differences remained or if the glycine group caught up once glycine presumably built some effect or if relapse occurred. In the enuresis RCT, what happened after 3 months? If homoeopathy was stopped, did children relapse or continue to improve? These questions remain unanswered.

### 4.6. Comparative Effectiveness and Integrative Approach

It’s informative to compare the magnitude of improvements seen here with those expected from conventional interventions. For insomnia, behavioural interventions (like bedtime fading and positive routines) are first-line and have a decent success rate (Meltzer & Mindell 2014 found behavioural interventions effective in ~80% of cases) [42]. The homoeopathic product might be working partly by simply calming the child at night (some ingredients like Cypripedium in material medica are used for sleepless infants). If it facilitates initiating sleep without needing ferberisation or lengthy battles, that’s a boon for parents. However, it should likely be used alongside good sleep hygiene—which any practitioner, homoeopathic or not, would advise. None of these studies suggest abandoning standard care; rather, they hint that homoeopathy can augment care.

From an integrative perspective, homoeopathic treatment is quite individualised and holistic, looking at the child’s physical and emotional state. For example, a child with enuresis who also has constipation and fears might receive a different remedy than one who is breezy and just a deep sleeper. This holistic matching might address subtle factors that a one-size drug like desmopressin does not. This could explain why some children respond to homoeopathy after failing standard therapy. Clinicians in integrative paediatrics might leverage this by referring refractory cases to homeopaths. However, the integrative field also demands evidence, and until now evidence was scant. Our review contributes by pulling together the best available evidence and showing that—while not definitive—it is suggestive of real benefits in certain contexts.

### 4.7. Implications for Future Research

Clearly, more studies are needed to build on these findings. Future trials should aim for larger sample sizes, multicentre participation, and rigorous blinding. Particularly:

A placebo-controlled trial for paediatric insomnia (perhaps in school-aged kids or adolescents) would fill a gap. One such trial, using *Coffea cruda* or another remedy for insomnia in teens, would be valuable to see if the positive outcomes seen in younger kids extend to older ages.

**Objective measurements**: incorporating actigraphy or wearable sleep tracker data could provide harder evidence of changes in sleep onset or duration, reducing reliance on parent reports.

**Mechanistic studies**: even small-scale, measuring physiological markers (e.g., stress hormones and EEG sleep architecture) in children on homoeopathy vs. placebo could either strengthen plausibility (if changes are seen) or not.

**Comparative studies**: e.g., homoeopathy vs. melatonin in ASD-related insomnia—since melatonin is now often used in neurodevelopmental disorders’ insomnia with some success [12,26,43,44,45], it would be interesting to see if homoeopathy can achieve similar outcomes without exogenous hormone use. This could be a non-inferiority trial design.

**Long-term follow-up**: do children treated with homoeopathy have more sustained resolution of their sleep problem compared to those on conventional meds or placebo? For enuresis, a 6–12 month follow-up would show if relapse rates differ.

**Health economics and acceptability**: Are families satisfied, and is quality of life improved by integrating homoeopathy? The Jong study’s high satisfaction hints that parents appreciated a solution with minimal side effects that worked [1]. Qualitative research might explore parent and child experiences with homoeopathic treatment for sleep issues—sometimes improved sleep can transform family life, which is an outcome not fully captured by scales.

### 4.8. Limitations of This Review

It’s important to acknowledge limitations in our own review process. We relied on the published literature and may have missed unpublished trials or those in languages other than English. We found one or two references to foreign studies (e.g., a Ukrainian journal article from 2012 on a homoeopathic syrup for sleep [1]), but these were older than our cutoff. We also encountered a recent narrative review (Kotian & Noronha 2024) which, while not systematically exhaustive, helped verify that we identified the major studies, as their table listed similar key references [32]. There is always a risk of publication bias, perhaps negative studies were performed but not published. The field of homoeopathy often faces this in both directions (some claim positive results are over-reported in obscure journals; others claim negative results are suppressed by sponsors). Our search did not find any clearly negative RCT in the last 10 years for paediatric sleep; it could be that there truly were none, or that investigators seeing no effect simply did not publish. This could skew our review towards an overly positive conclusion. However, given the relatively low number of trials conducted, it might just be that all who attempted such trials obtained at least mildly positive results.

Another limitation is that we did not perform a meta-analysis due to heterogeneity; thus, we could not produce an overall effect size. The evidence remains at the level of “multiple positive trials” rather than a consolidated quantitative measure. Each condition had at most one rigorous trial, so meta-analysis by subgroup was not feasible either (e.g., we cannot meta-analyse insomnia with only one real insomnia RCT). Our bias assessment was narrative and subjective; use of a standardised tool might yield slightly different gradings, but we attempted to be fair and transparent in our judgements.

This review itself and the body of evidence it summarises have several important limitations:

**Limited Number of Studies and Scope:** Only five studies met inclusion, reflecting the scarcity of rigorous research on this topic. The included trials covered a few conditions (insomnia in young children, bruxism, and enuresis). Significant gaps remain: we found no trials for other paediatric sleep disorders such as night terrors, chronic insomnia in adolescents, narcolepsy, or parasomnias like sleepwalking. Thus, our conclusions cannot be generalised to all sleep disorders in youth. The evidence is narrowly focused on certain age groups (e.g., under 6 for insomnia, under 12 for bruxism, mixed for enuresis). We acknowledge that we did not find data on adolescents specifically—a limitation given the question’s population scope; we highlight that as a gap for future research rather than a finding.

**Heterogeneity and Data Uncertainty:** The studies used different homoeopathic interventions and outcome measures, precluding meta-analysis. We had to synthesise qualitatively. For example, one study’s outcome was a composite score not used elsewhere, making cross-comparison difficult. We also note that some data were incompletely reported (e.g., the exact mean difference in bedwetting nights was not explicitly given; we inferred it from medians and *p*-values). We attempted to accurately represent results, but in places where data were lacking (e.g., standard deviations, etc.), we could not compute effect sizes. This introduces some uncertainty in the magnitude of benefit.

**Biases in Studies:** The methodological quality varied:

The PRISMA flow numbers in our review are somewhat imprecise. We transparently describe the selection process, but because of overlapping references and difficulty obtaining some records (one abstract in a conference proceeding was excluded due to insufficient data), our numbers are approximate. The included studies themselves had biases: Jong et al. was open-label (high risk of performance/detection bias) [1]; Saha et al. was uncontrolled (high risk of multiple biases) [30]. These biases could lead to overestimation of treatment effects. We did not formally use GRADE criteria, but informally, the evidence would likely be rated as low to moderate quality due to these limitations (imprecision, some risk of bias, and limited quantity).

There is also a potential conflict of interest note: the Jong et al. study was coauthored by employees of a homoeopathic manufacturer (DHU, Germany) [1]. While it was published in a reputable journal and likely peer-reviewed, industry sponsorship might bias study design or reporting. We did not have detailed info on funding for others; the Indian studies appeared government-supported (in a public hospital), which might mitigate commercial bias but could introduce an institutional bias favouring a positive result given they were conducted in homoeopathic institutions.

**Generalisability:** The cultural and healthcare context in which these studies were completed may limit applicability. For instance, the enuresis trial was in an Indian homoeopathy college hospital—patients there might differ from those in Western primary care. Acceptance of homoeopathy is higher in India; placebo effects might be larger if families strongly believe in it. Conversely, in a sceptical population, results might differ. The complex remedy trial was in Russia—glycine is a common supplement there, whereas elsewhere melatonin might be the comparator. So one must be careful extrapolating results to contexts where standard care differs.

**Data Gaps:** None of the studies reported longer-term follow-ups, so we do not know if the improvements are durable. Also, no studies formally assessed blinding integrity (e.g., asking parents if they thought their child received verum or placebo); it’s possible that subtle differences (or, in Jong’s trial, obvious differences since it was open) could have unblinded participants/investigators and biased outcomes.

**PRISMA flow and search limitations:** Our search might have missed some literature. For example, we did find references to a Chinese trial on homoeopathy for insomnia in ADHD (but it was not accessible), and there may be relevant studies published in languages like Spanish, French, or German we could not include due to language and time constraints (we limited ourselves to English as per inclusion criteria). We did not formally search grey literature beyond clinical trial registries, raising the risk of publication bias in our review.

**Analytical limitations:** We did not perform sensitivity analyses or subgroup analyses given the small number of studies. Ideally, one would examine, say, the effect in younger vs. older kids or individualised vs. complex approach differences, but we simply do not have enough data points.

Given these limitations, we emphasise that our conclusions are tentative. We acknowledge that the literature on paediatric homoeopathy for sleep is in an early stage, and our review highlights initial positive findings that need to be corroborated by further high-quality research. We have transparently noted where we had to estimate or rely on reported *p*-values without raw data (e.g., in summarising improvements). Furthermore, our review process itself was performed by a single group (the authors of this report) without external cross-checking, which could introduce error or bias in study selection or interpretation (though we attempted to be thorough and objective).

Any challenges with PRISMA flow data we faced (like reconciling multiple sources or unclear eligibility in borderline cases) have been described. For instance, we mentioned that literature is limited, and PRISMA counts reflect that limitation—only a handful of studies made it through, so the flow diagram is simple but underscored by the small yield.

**In summary**, while we have drawn evidence-based inferences, they rest on a slim evidence base, and both the existing studies and this review process have constraints that should temper how definitive our statements are. We advise readers and practitioners to view these results as encouraging but preliminary. The limitations enumerated here point to a clear mandate for more research and careful replication.

## 5. Conclusions and Future Research Directions

### 5.1. Conclusions

Homoeopathic treatments have shown a signal of potential benefit for certain paediatric sleep disorders in recent studies. In young children with behavioural insomnia, a homoeopathic complex remedy was associated with faster, more pronounced improvement in sleep onset and continuity compared to a common supplement, with excellent tolerability [1]. In children with sleep bruxism, a specific homoeopathic remedy (Melissa officinalis 12C) modestly reduced grinding severity, pointing to a possible non-invasive management tool [28]. For nocturnal enuresis, individualised homoeopathic prescribing led to a statistically significant reduction in bedwetting frequency relative to placebo, suggesting homoeopathy can augment standard enuresis care and help children achieve dryness sooner [29]. All interventions were very safe, with no serious side effects reported and high acceptability to patients and families.

However, these conclusions come with important caveats. The evidence base is still limited in size and scope. While positive, the findings need replication in larger, multicentre trials before homoeopathy could be recommended as a first-line therapy. At present, homoeopathic treatment might be considered as a complementary option for paediatric sleep disorders, especially in cases where conventional treatments are undesirable (e.g., parents who wish to avoid drugging their child to sleep) or ineffective. Physicians should ensure that any serious causes of a child’s sleep disturbance (like epilepsy, apnoea, or significant anxiety disorders) are ruled out or addressed, and if families choose homoeopathy, it should ideally be within an integrative framework that also emphasises proven behavioural strategies.

Our systematic review reveals that homoeopathy, when properly applied (matched to the child’s symptom profile or using a formula with traditional indications), may provide clinically relevant improvements in sleep for some children. These improvements, coupled with the minimal risk profile, indicate that further exploration of homoeopathy in this realm is warranted. It is a promising adjunct to conventional paediatric sleep management, but not yet a standalone replacement. We also conclude that parental satisfaction tends to be high when their child’s sleep improves without side effects [1], and this family-centric outcome is crucial—a good night’s sleep for a child often means a rested family, better daytime mood, and improved overall quality of life. In that sense, even moderate improvements (2 fewer wet beds a week, or falling asleep 15 min faster) can translate to meaningful relief in the household.

### 5.2. Future Research Directions

Building on the findings and limitations highlighted, we recommend several avenues for future research:

**Confirmatory RCTs:** Conduct larger-scale, blinded RCTs for the indications that showed promise. For instance, a multicentre RCT of individualised homoeopathy for enuresis in diverse settings (including outside of homoeopathic hospitals) would test reproducibility. Similarly, a placebo-controlled RCT for paediatric insomnia, perhaps comparing a homoeopathic remedy or complex to behavioural therapy and placebo, would be valuable.

**Expanded Conditions:** Investigate homoeopathy in other paediatric sleep disorders. Studies could examine if homoeopathic remedies can reduce the frequency of **nightmares or night terrors**, improve sleep in children with conditions like autism/ADHD (where insomnia is common), or even help with **delayed sleep phase** in teens. These are areas with high unmet needs where families might welcome gentle alternatives.

**Objective Outcomes:** Future trials should incorporate objective measurements (e.g., actigraphy watches to measure sleep duration and awakenings, polysomnography if feasible for specific questions like sleep architecture changes). This would add credibility and depth to outcome assessment, moving beyond subjective reports.

**Dose-Response and Remedy Specificity:** Research could explore optimal potencies and dosing schedules. For example, does Melissa 6C vs. 30C have a different effect on bruxism? Are multiple homoeopathic doses per day needed for insomnia, or would a single bedtime dose suffice (as is often completed with herbal sedatives)? Also, factorial trials could test whether the combination of remedies is better than a single remedy (the Tavares-Silva study hinted the combination was not better than single; more work could generalise that finding).

**Mechanism Studies:** Interdisciplinary research into how homoeopathic micro-doses might exert effects on the nervous system is encouraged. Laboratory models (like rodent sleep studies) using homoeopathic preparations of coffee, valerian, etc., could provide insight, as some earlier work in rats has suggested EEG changes with homoeopathic doses [1]. Understanding the mechanism (even if it is e.g., via nanoparticle presence or water structure theories) could help mainstream acceptance if effects are consistently observed.

**Longitudinal and Cessation Follow-up:** It would be useful to follow children after stopping homoeopathic treatment to see if improvements are sustained (homeopaths claim they often are, due to treating root causes). For example, in enuresis, track relapse rates 6 months after discontinuing the remedy versus placebo. If homoeopathy leads to more cures (permanent resolution), that’s a significant advantage.

**Comparative Effectiveness and Integration:** Future studies might compare homoeopathy head-to-head with standard treatments. For example, compare a homoeopathic complex vs. melatonin in children with insomnia for 4 weeks—see which yields equal or better sleep outcomes and fewer side effects. Or compare individualised homoeopathy vs. desmopressin for enuresis in a non-inferiority trial. Such studies would inform clinical decision-making directly. Additionally, studying homoeopathy in combination with standard therapy vs. standard therapy alone could quantify any additive benefit.

**Health Economics and Satisfaction:** If homoeopathy does prove beneficial, is it cost-effective? Studies could analyse cost savings from reduced need for other medications or fewer doctor visits for persistent sleep complaints. Patient-reported outcome measures (PROMs) and qualitative interviews with parents whose children underwent homoeopathic treatment can shed light on the less tangible benefits (empowerment, perception of child’s well-being, etc.).

**Broader sample diversity:** Ensuring future trials include a diverse population (in terms of ethnicity, socioeconomic status, and geographic region) will be important to see if the results hold broadly or if they are context-dependent. The first four studies included in Table 1 show relatively balanced proportions of male and female participants. Unfortunately, however, gender-specific analyses of this data are lacking. It should also be noted at this point that gender-related differences are also evident in sleep disorders in children and adolescents—if this aspect is taken into account, as has been shown, for example, in relation to gender- and age-related differences in obstructive sleep apnoea in children and adolescents [46].

**In conclusion**, our review provides evidence that homoeopathy may have a role in managing paediatric sleep disorders, but this role should be considered complementary and based on individual cases until stronger evidence emerges. The encouraging findings to date serve as a foundation upon which more definitive research can build. If future investigations confirm these outcomes and clarify how best to employ homoeopathy (which remedy, what dose, for which child), then homoeopathic treatment could become an accepted part of an integrative approach to paediatric sleep health—offering families a gentle, patient-centred option in the quest for restful nights.

Current epistemological advances in study planning and medical student training should be taken into account: critical and intersectional (or better still, transdisciplinary) thinking with retrospective examination of heuristic initial theses, gender aspects, life course health, context variables and criteria for individualised, patient-related precision medicine. We consider this appeal to be very important, as although the Flexner Report of 1910 led to fundamental improvements in the quality of medical research, training and care in the Western world, it was too one-sidedly oriented towards group benefits at the expense of taking into account the individual characteristics of each patient [47,48,49,50,51,52,53].

This means that medical history, age, gender, context-related variables, and clinical and psychological findings should not be sacrificed on the altar of diagnostic equipment, molecular genetics and epigenetics. Critical thinking is the order of the day, i.e., heuristic initial hypotheses that appear innovative should be analysed retrospectively in order to reduce error rates in differential diagnostics and study planning. In our view, the classic conflict between ‘conventional medicine’, which sees itself as evidence-based medicine, and complementary medicine approaches, including homoeopathy, can only be resolved through holistic thinking that takes into account the above-mentioned requirements in training, research and patient care.

Overall, it is a welcome and serious trend that homoeopathic therapies are now being put to the test of evidence-based medicine and that corresponding guidelines have been published [13,14]. This focus on such open-ended studies should also be specifically developed in the training of medical students, as it is not an automatic process: “Porzsolt et al. showed that medical students are more motivated to engage with the criteria of evidence-based medicine if they have examined sick people relatively often themselves and are willing to think openly about their own educational background. Jacobsen and Jacobsen have described this as the inner willingness to change perspectives in the sense of ‘framing knowledge’.

In the context of evidence-based medicine, this involves the following five steps:

(1) Formulating questions
(2) Researching to gather relevant information
(3) Evaluating existing external findings
(4) Combining external findings with one’s own experiences and the values of the patients concerned
(5) Evaluating the process initiated).” [54].

From an epistemological point of view, further studies should take into account that it is not only a matter of interdisciplinary thinking, but that transdisciplinary discourses are deliberately taken up. This means that other disciplines should not only be cited and formally taken into account but also addressed in terms of content, and that methodological experiences from other disciplines should also be involved. In this sense, medicine can benefit from the epistemological foundations and methodological resources of sociology, evolutionary history research, behavioural biology, history, comparative cultural studies and anthropology.

From a medical history perspective, it is interesting to note that Hahnemann’s homoeopathy, with its roots in Germany and Paris [15,16,55], spread rapidly via doctors from Europe to the United States and India, where it became highly institutionalised [56,57]. The Flexner Report from 1910 mentioned above led to the closure of numerous homoeopathic institutions in the USA and the Western world and to reservations about homoeopathy in European countries that continue to this day [58]. In India, on the other hand, homoeopathy continues to flourish to this day because this gentle and inexpensive therapy appeared to be a welcome contrast to the arrogance of English colonial representatives towards the natural medicine established in India [56]. From today’s perspective, holistic approaches are necessary in the best sense of the word, in which the well-being of each individual patient is at the forefront [59]. Evidence-based medicine, research and training are fundamental pillars of this approach [13,60,61]. In conceptual terms, it should be noted that in India, homoeopathy is not only understood as the administration of globules but is also classified as an umbrella term for holistic approaches to differential diagnosis and therapy, which include consistent proof of effectiveness according to the criteria of evidence-based medicine, advocacy models that respect the patient’s perspective, plus active professional consultation and criteria of individualised precision medicine [17,18,19,21,22,62,63,64,65,66].

## Figures and Tables

**Table 1:** Key characteristics of included studies *.

Author(s) (Year)	Country	Study Design	Sample Size	Age Range	Sleep Disorder Type	Outcome Measures (Key)
Jong et al. (2016) [1]	Russia (multi-centre)	RCT (open- Label and active control)	N = 179 (89 vs. 90)	1–6 years (52 m/37 f verum; 51 m/39 f placebo) **	Insomnia and restlessness (≥1 month)	Total complaints severity score; time to sleep onset; night awakenings; Integrative Medicine Outcome Scale (IMOS); patient/parent satisfaction (IMPSS)
Tavares- Silva et al. (2019) [28]	Brazil	RCT (crossover, triple-blind, and placebo-controlled) ***	N = 52 (crossover)	Mean 6.6 ±1.8 years (range 5–12; 27 m/25 f in these 4 groups) **	Possible sleep bruxism (teeth grinding)	Parental VAS for bruxism severity; sleep diary (sleep quality); Trait Anxiety Scale for Children (TAS); side effects log
Akram et al. (2025) [29]	India	RCT (parallel, double-blind, and placebo-controlled)	N = 140 (70 vs. 70)	5–16 years (33 m/37 f verum; 28 m/42 f placebo) **	Nocturnal enuresis (bedwetting)	Frequency of bedwetting (episodes/week); Paediatric Quality of Life (PedsQL) child and parent report; cure rate (dry nights); adverse events
Saha et al. (2018) [30]	India	Observational (pre–post single-arm)	N = 34	5–18 years (17 m/17 f) **	Nocturnal enuresis (bedwetting)	Nocturnal enuresis severity score (custom 0–15 scale counting nights and intensity); measured at baseline, 2 months, and 4 months
Harrison et al. (2013) [31] (Excluded from final)	UK (for context)	RCT (double-blind and placebo-controlled)	N = 46	7–12 years (all male)	Psychophysiological insomnia (PI)	Pre-Sleep Arousal Scale (PSAS); sleep diaries; global improvement (note: older study, outside 10-year range)

(*) Harrison 2013 is shown for context but was not included in the quantitative synthesis due to the publication date being >10 years old; it reported positive trends in a complex remedy for childhood insomnia [31,32]. (**) m = boys; f = girls. (***) A key strength of this study is that the active ingredient content in all three verum groups was verified and documented by laboratory analysis.

**Table 2:** Homoeopathic intervention details. Potencies are on the centesimal scale unless noted. “Individualized” indicates remedy and dosing were tailored to each patient by a homeopath, rather than a one-size-fits-all regimen. (Harrison 2013 data shown for context of prior work; not included in analysis as per criteria.).

Author(s) (Year)	Homoeopathic Medicine Name(s)	Potency & Dosage/Regimen	Treatment Duration	Control/Comparator (If Any)
Jong et al. (2016) [1]	ZinCyp-3-02 (complex of Cypripedium D4, Magnesia carb. D10, Zincum valerianicum D12)	1 tablet 4 times daily (dissolved in water for <3 yrs.)	4 weeks (28 days)	Glycine 100 mg tablet, 4× daily (active comparator)
Tavares-Silva et al. (2019) [28]	*Melissa* *officinalis* 12C; Phytolacca decandra 12C; (also combined MO + PD)	5 globules once nightly (implied); crossover: each child received 30 days of each regimen, with 15-day washouts	4 × 30-day phases (crossover, total ~5 months)	Placebo (matching globules) during one phase of crossover
Akram et al. (2025) [29]	Individualised homoeopathic remedy (e.g., Sulphur, Calc. phosphorica, Calc. carbonica, etc., chosen per case)	Potency varied (commonly 30C/200C); given typically once or twice daily; the remedy could be adjusted at monthly visits	3 months (with monthly follow-ups)	Placebo globules (identical look/taste) + standard care advice (fluid restriction, alarms, etc.)
Saha et al. (2018) [30]	Individualised homoeopathic medicine (most common: Kreosotum in 26% of cases, others like Sulphur, Nux vomica, etc.)	Potency 30C or 200C (typical), dosage individualised (e.g., one dose nightly); adjustments made at 2-month and 4 month visits if needed	4 months total (evaluation at 2 and 4 months)	None (no control group in this single-arm trial)
Harrison et al. (2013) [31] (not in final synthesis)	Complex homoeopathic blend for insomnia (included Coffea 30C, etc.)	2 tablets at bedtime (per author’s report)	4 weeks	Placebo tablets (double-blind)

**Table 3:** Summary of bias assessment for included studies. Each study was reviewed for major potential biases. RCTs generally had low risk in randomisation/blinding except where noted; the open-label trial and uncontrolled study carry higher risk. Overall, evidence quality is moderate given some methodological limitations, which are considered in interpreting results.

Author(s) (Year)	Domain Assessed	Bias Assessment Method (Narrative)	Judgement/Concerns
Jong et al. (2016) [1]	Blinding (Performance/Detection)	Evaluated as an open-label design; the outcome (symptom score) was rated by investigators/parents aware of the treatment	High risk—lack of blinding could inflate perceived improvements in the homoeopathy arm (observer expectancy bias).
Tavares-Silva et al. (2019) [28]	Selection Bias (Randomisation)	Reviewed the randomisation procedure in the triple-blind RCT; assignment was random and crossover counterbalanced (each child as their own control)	Low risk—proper random sequence and allocation; crossover design with each child receiving all treatments reduces between-group differences.
Tavares-Silva et al. (2019) [28]	Carryover Effect (Crossover)	Analysed washout adequacy (15 days) and period effect; no significant period/order effects reported by authors	Some concerns—a 15-day washout may not fully prevent carryover, but it is unlikely to be a major issue given that homoeopathic 12C likely has a transient effect; overall design is robust.
Akram et al. (2025) [29]	Performance/Detection Bias	Double-blind RCT: patients, prescribers, and evaluators blinded; outcomes (bedwetting frequency) objectively counted by parents, likely unbiased by group	Low risk—blinding was maintained; the placebo was identical to the verum, minimising expectation bias. (Slight risk if parents guessed treatment due to improvement, but unlikely).
Akram et al. (2025) [29]	Attrition Bias	Monitored dropouts (only 4 total dropouts, evenly split); used intention-to-treat analysis for primary outcome	Low risk—minimal attrition and no differential loss between groups; results robust.
Saha et al. (2018) [30]	Confounding (Study Design)	Observational one-group pre-post; no control for placebo effect or maturation; baseline to outcome comparison only	High risk—improvement could partly reflect spontaneous resolution or parent perception changes; positive results must be interpreted cautiously without a control group.
All studies (general)	Reporting Bias	Checked outcomes vs. methods for each study; all predefined outcomes reported, no selective omission noted (e.g., no significant diaries in Tavares’s study were acknowledged)	Low risk—no evident selective reporting. Publication bias in the field is possible (negative trials may be unpublished), but within the included studies, reporting was transparent.

## Data Availability

The original contributions presented in the study are included in the article, further inquiries can be directed to the corresponding author.
